# Controlling the Diameter of Single-Walled Carbon Nanotubes by Improving the Dispersion of the Uniform Catalyst Nanoparticles on Substrate

**DOI:** 10.1007/s40820-015-0050-8

**Published:** 2015-07-23

**Authors:** Junjun Chen, Xiangju Xu, Lijie Zhang, Shaoming Huang

**Affiliations:** Nanomaterials and Chemistry Key Laboratory, Wenzhou University, Wenzhou, 325027 People’s Republic of China

**Keywords:** Single-walled carbon nanotube, Diameter control, Chemical modification, Chemical vapor deposition, Catalyst nanoparticles

## Abstract

To have uniform nanoparticles individually dispersed on substrate before single-walled carbon nanotubes (SWNTs) growth at high temperature is the key for controlling the diameter of the SWNTs. In this letter, a facile approach to control the diameter and distribution of the SWNTs by improving the dispersion of the uniform Fe/Mo nanoparticles on silicon wafers with silica layer chemically modified by 1,1,1,3,3,3-hexamethyldisilazane under different conditions is reported. It is found that the dispersion of the catalyst nanoparticles on Si wafer surface can be improved greatly from hydrophilic to hydrophobic, and the diameter and distribution of the SWNTs depend strongly on the dispersion of the catalyst on the substrate surface. Well dispersion of the catalyst results in relatively smaller diameter and narrower distribution of the SWNTs due to the decrease of aggregation and enhancement of dispersion of the catalyst nanoparticles before growth. It is also found that the diameter of the superlong aligned SWNTs is smaller with more narrow distribution than that of random nanotubes.

## Introduction

Since the first report by Iijima in 1991 [1], carbon nanotubes (CNTs) have attracted tremendous attentions both for fundamental research and potential applications in many fields [2–4]. Especially, the single-wall carbon nanotubes (SWNTs) are much more interesting for scientists due to their unique one-dimensional structure, mechanical, and electrical properties [5]. SWNTs consist of a single graphite sheet seamlessly rolled into cylindrical tube. They can be grown by several methods including arc discharge [6], laser ablation [7], and chemical vapor deposition (CVD) [8]. Among these methods, CVD is the most promising method for low-cost and large-scale production of highly pure nanotubes. SWNTs can be grown in high temperature using carbon-containing molecules such as methane, ethane, or carbon monoxide [9] as carbon sources with the help of catalytic particles, usually metal nanoparticles (NPs) such as Fe, Co, Ni, and their alloys. In most cases, only SWNTs with different chiralities can be generated, this is not beneficial for their applications particularly for electronic devices application because the electrical properties of the SWNTs depend not only on their structure (chirality) [10, 11] but also on their diameter [12]. Therefore, how to take effective control of the chirality and diameter is still a goal of current research [13–25]. Basically, the diameter of SWNTs depends on the size of the catalyst. The catalyst can be pre-obtained by either chemical method (nanoparticles in solution) or physical methods (such as arc plasma or sputtering deposition or thermal treatment of thin film of catalyst on substrate). How to prepare uniform catalyst size is the key for controlling the diameter of SWNTs. In the past decades, many efforts have been put on to control the catalyst size for the control of the SWNTs diameter. For example, Dai and Pfefferle got uniform diameter SWNTs using polyamidoamine dendrimer [26] or MCM-41 [27] as catalyst carriers, respectively. Hyon et al. reported one way to control the diameter of the SWNTs by Langmuir–Blodgett film [28]. Jie used identical metal-containing molecular nanoclusters as catalysts and investigated the relationship between the diameter of SWNTs and carbon feeding [29, 30]. Size-separated ferritin-based Fe catalyst [31], inorganic oxides nanoparticles with high melting point such as SiO_2_, TiO_2_, Al_2_O_3_ are also used as catalysts for growing SWNTs [32–34]. Arc plasma deposition [35] or floating catalyst method [36] are applied to control the size and density of the catalysts for growing either vertically aligned SWNT forest or narrowing the SWNT diameter distribution. Although significant progress has been made, to develop a facile technology to control accurately the diameters of SWNTs and to understand the relationship between the diameters of SWNTs and the catalyst size still remains as a challenge. It is believed that uniform NPs individually dispersed on the substrate before CNTs growth at high temperature is one of the keys for controlling the diameters of the SWNTs. However, catalyst NPs particularly metal NPs tend to aggregate at high temperature. In this article, we report a facile approach to control the diameter of SWNTs by improving the dispersion of the Fe/Mo NPs on silicon wafers with different surface properties by chemical modification with 1,1,1,3,3,3-hexamethyldisilazane (HMDS).

## Experimental

**Scheme 1:**
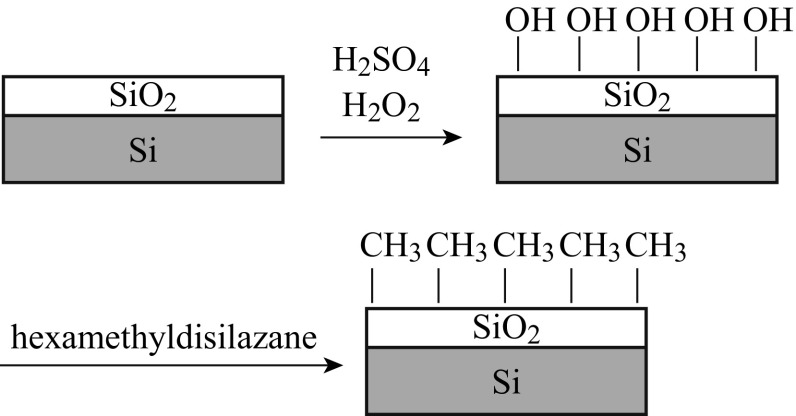
Schematic representation of the chemical modification on substrate surface

In our experiment, silicon wafer with 200-nm thick silica on top as substrate was used as substrate. The substrates were modified by 1,1,1,3,3,3-hexamethyldisilazane. The overall synthetic procedure was described in Scheme 1. To start with, all wafers were first cleaned by piranha solution (a mixture of 98 % H_2_SO_4_ and 30 % H_2_O_2_ at 7:3 v/v) for about 30 min, followed by thorough rinsing with deionized water for several times. Then, 10 mL of HMDS was added into a steel chamber (300 mL in volume). The wafers were then put into the chamber at room temperature and 150 °C for different times, respectively (without modified, modified for 1 min at room temperature, and modified for 30 min at 150 °C). The modification of the substrate surface was monitored by contact angle measurement using water as (EASYDROP Contact Angle Measuring System, Krüss Company).

The uniform Fe/Mo NPs were synthesized by thermal decomposition of Fe(CO)_5_/Mo(CO)_6_ under the protection of the surfactant according to the Ref. [37]. The dispersion of the Fe/Mo NPs was achieved by dipping the modified and without modified wafers into the hexane solution containing Fe/Mo NPs. After the solvent was evaporated, the dried wafers were heated in hydrogen (H_2_) at 900 °C. When the furnace was cooled down to room temperature, the distribution of particles was characterized by atomic force microscopy on tapping mode (AFM: Nano Scope III).

CVD method was used for SWNTs growth. Typically, the wafer with the catalyst was put in a quartz tube. Then the quartz tube was put into a tube furnace and heated in argon (Ar). Then after the furnace was heated up to 900 °C under the mixture of Ar and H_2_, Ar was turned off and methane was introduced into the tube (ratio of CH_4_/H_2_ = 4:1). After 15 min, methane and H_2_ were turned off and the quartz tube was cooled to room temperature under the Ar. The samples were characterized by AFM and scanning electron microscope (SEM: FEI NanoSEM at 10 kV) and Raman spectroscopy (Dilor Raman spectrometer with triple spectrograph at 514.5 nm excitation wavelength with an Ar-ion laser source at a power of 120 mW). The diameter of the SWNTs was measured by AFM.

## Results and Discussion

**Fig. 1:**
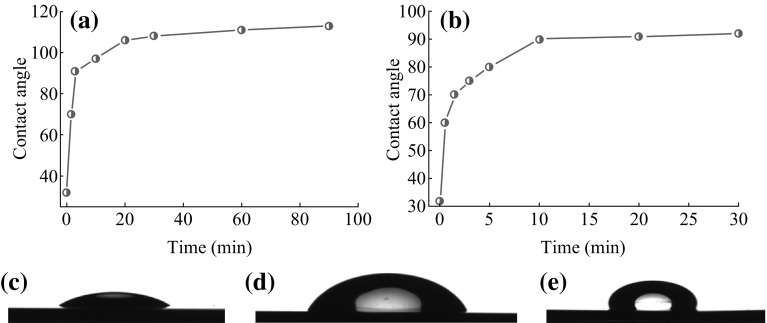
The relationship between the reaction temperature and the contact angle of the substrate modified by HMDS (**a**) at room temperature, (**b**) at 150 °C. The photo pictures of the water droplet on the bare substrate (**c**) modified with HMDS at room temperature for 1 min (**d**) and 150 °C for 30 min (**e**)

**Fig. 2:**
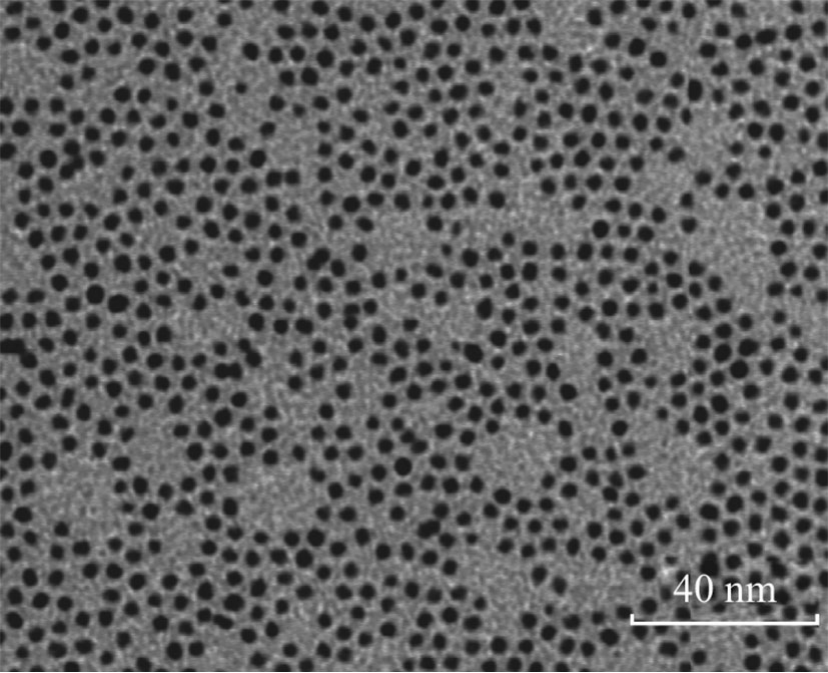
TEM image of Fe/Mo NPs

**Fig. 3:**
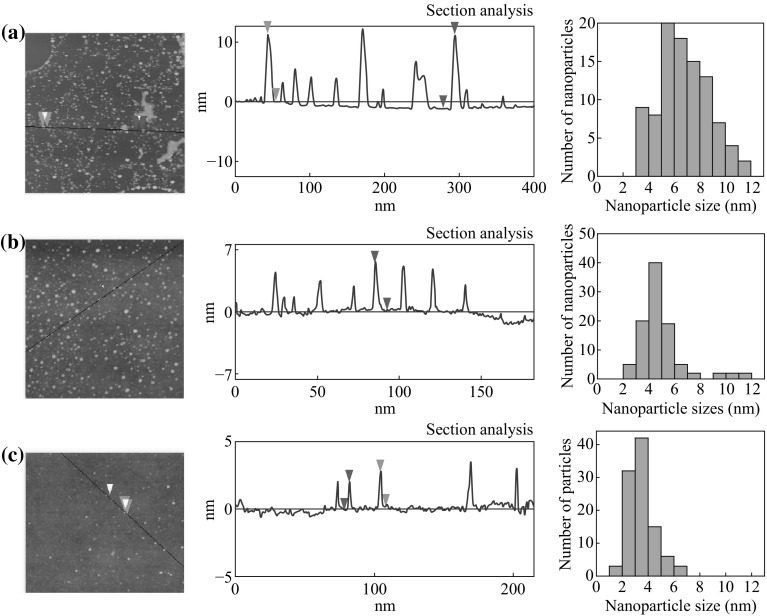
AFM images, height measurement and diameter distribution of the Fe/Mo NPs on different substrate surfaces (**a**) bare substrate, modified by HMDS for 1 min at room temperature (**b**) and 150 °C for 30 min (**c**)

At room temperature, the contact angle of the bare Si/SiO_2_ substrate was measured to be about 32° (Fig. 1a). But after the substrate was exposed to HMDS in a steel chamber, the contact angle of the substrate was found to increase with the extension of time (Fig. 1a). When the contact angle reached to 90°, it would not change with the time. However, when steel chamber was put in the oven at 150 °C, the contact angle of the substrate will change with the time until the contact angle reaches about 112° (Fig. 1b). In order to study the relationship of the surface property of the substrates and the dispersion of the catalyst NPs, three substrates were selected for comparison: (a) bare substrate (contact angle is 32°); (b) the substrate modified for 1 min at room temperature (contact angle is 71°); (c) the substrate modified for 30 min at 150 °C (contact angle is 110°). The picture of the water droplet is given in Fig. 1c–e, respectively. The catalyst used for the growth of SWNTs is uniform Fe/Mo NPs wrapped by organic surfactant in the n-heptane. The diameter of the NPs was measured to be 3.8 ± 0.8 nm according to TEM observation (Fig. 2). After dispersion of the solution containing Fe/Mo NPs on the substrate, the substrate was treated under Ar at 300 °C to remove the organic surfactant. Figure 3a shows AFM images and height measurement of the Fe/Mo NPs after dispersion on the bare substrate. It was easy to note that some NPs aggregated because of the hydrophilic surface of the bare wafer. The corresponding statistical size distribution of the NPs ranging from 3.0 to 12.0 nm is given in Fig. 3a. The main size was 5.0–9.0 nm, indicating the aggregation of the Fe/Mo NPs on the bare substrate surface. The dispersion of the Fe/Mo NPs on the substrate can be improved a lot after the modification by HMDS because the substrate became hydrophobic. When the contact angle of the substrate is 71° and 110°, the main size of the Fe/Mo NPs is 3.0–6.0 nm (Fig. 3b) and 2.0–5.0 nm (Fig. 3c), respectively. From this result, it is obvious that the aggregation degree of the Fe/Mo NPs on the substrate surface can be decreased by modifying the substrate from hydrophilic to hydrophobic. As the size of the Fe/Mo NPs is 3.8 ± 0.8 nm, most of the NPs can be dispersed on the substrate separately when the surface becomes fully hydrophobic.

**Fig. 4:**
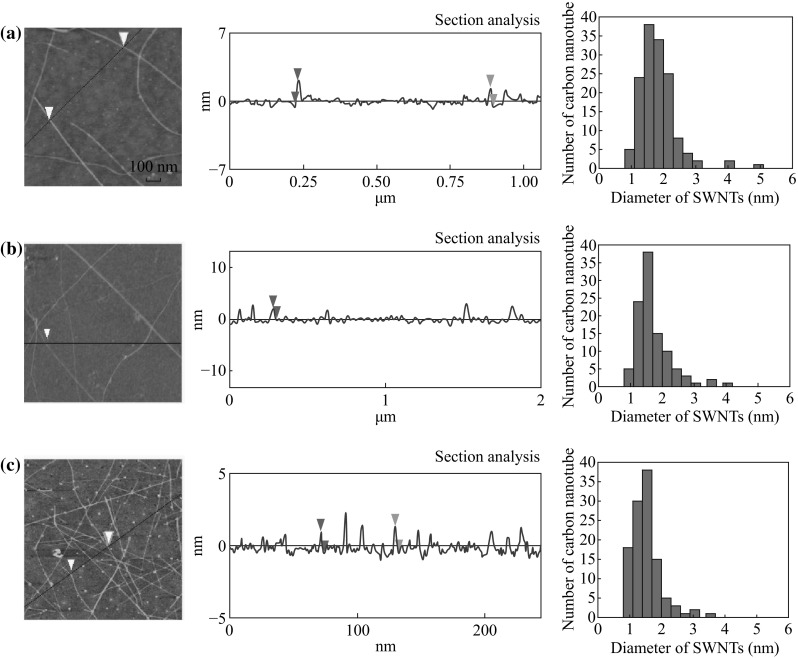
AFM images, height measurement and diameter distribution of SWNTs on different substrates (**a**) bare substrate, modified by HMDS for 1 min at room temperature (**b**) and 30 min at 150 °C (**c**)

It is well known that the diameters of CNTs depend strongly on the catalyst size. Uniform Fe/Mo NPs have been widely used as active catalyst for the growth of SWNTs on substrate [9]. Changing the wettability of the SiO_2_/Si substrate has been applied to control the density of the nanotubes and pattern of the nanotubes [38]. By improving the dispersion of the uniform Fe/Mo NPs on the substrate surface a better control for the uniformity of the SWNTs diameters could be expected. Figure 4 presents the AFM images and the diameter distribution of the SWNTs growing on the substrates with or without chemical modification using methane as carbon source. Figure 4a is the AFM image and height measurement of an individual SWNT and the diameter distribution of the SWNTs, as well on the bare substrate. From the diameter distribution, the main diameters of the nanotubes was 0.8–3.3 nm (the main diameter of the SWNTs is 1.75 nm). When the substrate was modified by HMDS, the diameters of the SWNTs were found to decrease and the size distribution became narrow. Figure 4b, c gives the AFM images and the height of the individual SWNT and size distribution. The main diameters of the SWNTs is 0.8–2.8 nm (the main diameter of the SWNTs is 1.50 nm) for the substrate modified by HMDS for 1 min at room temperature (Fig. 4b) and 1.1–2.0 nm (the main diameter of the SWNTs is 1.46 nm) for the substrate modified by HMDS for 30 min at 150 °C, respectively. This results indicate that the hydrophobic surface will reduce the aggregation and improve the dispersion of the catalytic NPs on the surface because the uniform Fe/Mo NPs was dispersed in hydrophobic solvent (n-heptane), thus reducing the diameter of the nanotubes and narrowing the diameter distribution. This approach as-demonstrated is simply changing the hydrophobicity, matching the solvent in which the catalyst NPs are dispersed. This method should be a simple and facile one compared with other methods applied in controlling the diameter of SWNTs [35, 39–42].

**Fig. 5:**
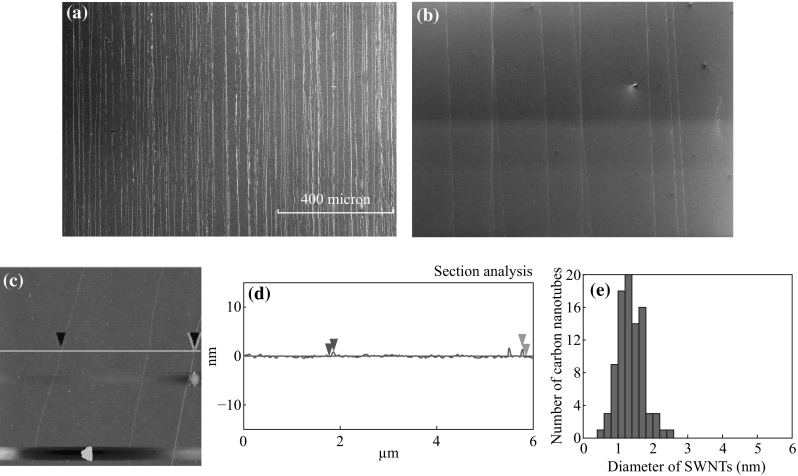
SEM images (**a**, **b**) and AFM image (**c**), height measurement (**d**) and diameter distribution of the long-aligned SWNT arrays on the substrate modified by HMDS for 1 min at room temperature (**e**)

As we have demonstrated that when “fast-heating” CVD is used superlong well-aligned SWNTs arrays can be generated and “kite mechanism” has been proposed to describe how the long SWNTs can grow and how they can be aligned (gas flow guidance) [43–45]. What is interesting to find is that the diameter of the long-oriented SWNTs was smaller with more narrower distribution than that of random short SWNTs. Figure 5a, b is the SEM images of the superlong well-aligned SWNT arrays, and Fig. 5c–e is the AFM image, height measurement, and diameter distribution of the long-aligned SWNTs on the substrate modified by HMDS for 1 min at room temperature. The diameter of the SWNTs ranges from 0.5 to 2.5 nm (the main diameter is 1.26 nm). Comparing with the random SWNTs (Fig. 4) and well-aligned SWNT array (Fig. 5c–e), it was easy to note that the diameter of the aligned SWNTs is larger than that of random one and the diameter distribution of the aligned SWNTs is much narrower that that of the random nanotubes on bare substrate (Fig. 4a), and even narrower than that of the nanotubes on modified substrate (Fig. 4b, c). As we have demonstrated the growth of the superlong nanotubes under “fast-heating” CVD condition is tip-growth mechanism (kite mechanism) and the orientation of the nanotubes is controlled by the gas flow [44]. The “fast-heating” process limits the aggregation of the catalyst NPs on the substrate and the catalyst with small size should be relatively easy to grow in tip-growth mechanism because of the relative weak interaction with the substrate surface. Furthermore, the aligned SWNTs growth in the gas flow also makes the nanotubes much cleaner than that of the random nanotubes which locates the catalyst NPs area.

## Conclusions

In this study, we successfully improved the Fe/Mo NPs dispersion and reduced their aggregation on substrate surface by modifying the substrate from hydrophilic to hydrophobic. Different diameters and distribution of SWNTs can be generated by using different size NPs based on CVD. For fully hydrophobic surface, Fe/Mo NPs can be possibly separate individual dispersion, and high-yield SWNTs with narrow size distribution SWNTs can be obtained. It is also found that the diameter of the superlong aligned SWNTs is smaller with more narrow distribution than that of random nanotubes due to the tip-growth mechanism for oriented nanotubes. Results further confirm that the diameter of SWNTs has a closed relationship with the size of catalyst and also provide an effective way to control the diameter and size distribution of SWNTs by improving the dispersion of the uniform catalyst NPs on surface.
